# Clinical Governance to Enhance User Involvement in Care: A Canadian Multiple Case Study in Mental Health

**DOI:** 10.34172/ijhpm.2020.208

**Published:** 2020-11-07

**Authors:** Nathalie Clavel, Marie-Pascale Pomey

**Affiliations:** ^1^Ingram School of Nursing, McGill University, Montreal, QC, Canada.; ^2^McGill University Health Centre, Montreal, QC, Canada.; ^3^Department of Health Policy, Management and Evaluation, School of Public Health, University of Montreal, Montreal, QC, Canada.

**Keywords:** User Involvement, Clinical Governance, Managerial Practices, Clinical Practices, Patient-Reported Experience, Mental Health

## Abstract

**Background: **Individuals with serious mental illness face challenges in managing their health, leading to the need for integrating their needs and preferences in care decisions. One way to enhance collaboration between users and providers is to improve clinical governance; a shared responsibility between managers and providers, supported by healthcare organizations (HCOs), policies, and standards. We applied the concept of clinical governance to understand (1) how managers and providers can enhance the involvement of users in mental health, (2) the contextual and organizational factors that facilitate user involvement in care, and (3) the users’ perceptions of their involvement in care.

**Methods:** We conducted two, in-depth case studies from two clinical teams in Canada offering outpatient care for users with acute mental illness. A total of 25 interviews were carried out with managers, and four focus groups were held with providers. A measure of patient-reported experience was used to evaluate the users’ perceptions of their involvement in care.

**Results: **The providers used two methods to involve users in the care planning process: encouraging users to identify their life goals and supporting them to define recovery-oriented objectives. To encourage the adoption of collaborative practices, the managers used various practices such as revising care protocols, strengthening providers’ knowledge of best practices and integrating peer-support workers (PSWs) in the team. Compliance with organizational and external commitments/requirements for user involvement, access to specific training and the institutionalization of a culture promoting user involvement facilitated the adoption of collaborative practices. We found that mental health teams that adopt recovery and collaborative practices with users show a high degree of user-perceived involvement in care.

**Conclusion: **This is the first study to apply the concept of clinical governance to understand how managerial and clinical practices, and other organizational and contextual factors, can enhance the involvement of mental healthcare users.

## Background

**Key Messages**

** Implications for policy makers**

- Collaborative practices with users, including the integration of a recovery approach and peer-support workers (PSWs), should be encouraged and supported within mental health teams. In this regard, the roles of managers at the clinical level are particularly important. Their roles need to be supported by policies and reference frameworks elaborated by healthcare organizations (HCOs) and authorities with regard to user involvement in mental health.
- Access to specific training on user involvement in mental health is crucial to develop the knowledge and skills of providers on recovery and collaborative practices with users. It is important that healthcare authorities make available training for mental health teams on user involvement in care to address best practices and specific challenges in this area.
- User involvement initiatives in quality improvement could be introduced in mental health teams to develop a culture of user participation among providers and managers and to foster the adoption of collaborative practices with users in their care decisions. Healthcare authorities have a key role to encourage and support HCOs and teams to involve users in quality improvement activities and structures.

** Implications for the public**

Users with serious mental illness face challenges in managing their health and treatments. This has led to the need for integrating users’ needs, expectations and preferences in care decisions with support from providers. This study seeks to understand: (1) how mental health teams, including providers and managers, can enhance user involvement in their care; (2) which factors, at the levels of healthcare organizations (HCOs) and healthcare systems can foster mental health user involvement in care; and (3) what are the perceptions of mental health users about their involvement in care. By looking at the practices and factors that enhance the involvement of mental health users in their care decisions, the findings of this research may ultimately help users to gain control over their health and treatments to improve their quality of life.

User involvement has become a priority for healthcare organizations (HCOs), governments, and accreditation bodies seeking to improve the quality of care. A growing body of evidence suggests that involving users who have chronic conditions can help reshape their care and treatments in ways that are more acceptable for users, ultimately resulting in improved clinical outcomes.^1-5^ Users with a serious mental illness often face challenges in managing their health and treatments, resulting in worse clinical outcomes like exacerbation of symptoms, re-hospitalization, and poor benefits of treatment.^6^ International publications like the 2001 World Health Report,^7^ the Lancet series on global mental health,^8^ and the United Nations Convention on the Rights of Persons with Disabilities^9^ call for actions to enhance the participation of people with mental health conditions and to respect their rights as citizens. These clinical and rights-based arguments have led to the need for collaboration between users and providers, where the needs, preferences, and experiences of users are included in their care decisions.

 User involvement can be defined in several ways but it is broadly recognized as the process or activities that enhance the participation of users in their health and treatments.^10^ The involvement of users in their care can be examined as a spectrum from low to highly collaborative models (ie, information, consultation, collaboration, partnership), which are associated with different degrees and modalities of user involvement.^11,12^ The paternalistic model that has dominated care since the late 1980s excludes users from decision-making by minimally informing them about their diagnoses and treatments.^13^ Introduced in the 1990s, patient-centred care seeks to integrate the needs, preferences, and values of users into their care decision-making, though it primarily focuses on the practices of providers without defining the users’ contribution to care decisions. In recent decades, collaborative approaches, such as shared decision-making have encouraged users to take part in their care decisions.^13-18^ Shared decision-making is defined as a process where decisions are made jointly by the user and a healthcare professional.^18^ More recently, the patient partnership approach has considered users as full-fledged members of health teams and seeks to involve users within interdisciplinary meetings.^12,19,20^ In mental health, a recovery-oriented care movement has emerged that prioritizes users’ autonomy, empowerment, respect, and shared decision-making.^6,21^ Recovery is often referred to as a process or guiding principle that focuses on regaining a meaningful life for people living with persistent mental health symptoms.^22^ Shared decision-making aims to facilitate user participation in the planning of care and it is a key component for a successful recovery process.^23,24^

 In Canada, accreditation bodies and governments have recently defined new guidelines, standards, and policies that make user involvement a core dimension of healthcare delivery. In 2015, Accreditation Canada integrated the involvement of users and their families in all of the care and services standards.^25^ In direct care, all users have to be involved in developing their care plan. In parallel, the action plan in mental health 2015-2020 of the Quebec Ministry of Health and Social Services (MSSS) makes the recovery approach one of the guiding principles of care and services in mental health.^26^

 Consequently, mental health settings are increasingly encouraged to enhance user involvement in care decisions. In that context, it is particularly interesting to understand how user involvement is integrated into mental health teams and supported by HCOs. User involvement can result from the efforts of providers and from clinical governance, as a shared responsibility of both managers and providers, supported by the HCOs and influenced by health policies and standards. Clinical governance can be defined as the set of processes, systems, and efforts to ensure the deployment of best clinical practices for quality improvement purposes.^27-29^ Clinical governance is relevant and can be operationalized to understand how managers and providers enhance user involvement, which is a core dimension of the quality of care. To this end, we ask three specific questions:

1. How do managers and providers contribute to enhancing user involvement in care using key practices?
2. What contextual and organizational factors facilitate user involvement in care?
3. What are the users’ perceptions of their involvement in care? Are user involvement-oriented practices associated with a high degree of user-perceived involvement in care?

###  Conceptual Framework

 To answer questions 1 and 2, we relied on literature that defines clinical governance as a set of systems, processes, and practices that aims to improve quality.^27-31^ We focused on two levels of clinical governance: (1) “governance by”^30^ or “proximity governance,”^32^ which refers to the practices of managers and providers at the clinical level to enhance user involvement in care; and (2) “governance of”^30^ or “system governance,”^32^ which is the system for accountability at a higher level (HCO and policy system) that supports the efforts of managers and providers to deploy user involvement in care. We chose four main dimensions and draw from several studies on clinical governance.^30-34^ The dimensions are present at both levels of governance^30^:

*Accountability:* These mechanisms ensure that user involvement is achieved with regards to the goals and objectives set by the organization or the system level.

*Audit:* These methods and tools ensure that quality is based on best practices or standards related to user involvement.

*Training:* Training activities are performed to update the knowledge and skills of providers for evidence-based practices on user involvement.

*Culture:* Actions are carried out to encourage the development of a culture of user involvement within teams and HCOs (Figure).

**Figure:**
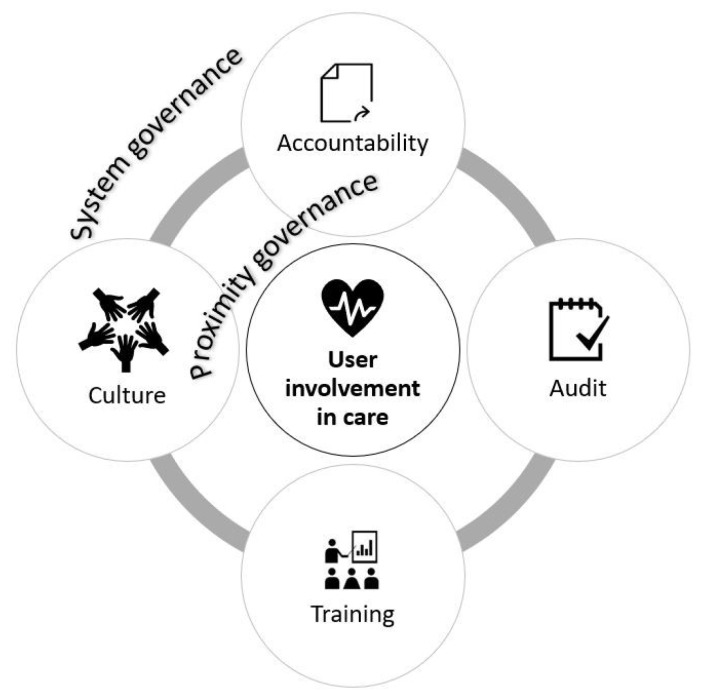
Conceptual Framework.

 To answer question 3, we focused on evaluating the users’ perceptions of their involvement in care.

## Methods

###  Case Study Design 

 We conducted in-depth case studies^35^ from two clinical teams in two different mental health settings situated in different HCOs in Canada (Quebec). A multiple-case design was chosen to develop a more in-depth understanding of the practices and factors that can enhance mental health users’ involvement in care, compared to a single case design.^36^ The longitudinal design of the research (2015 to 2017) enabled us to study how providers and managers enhance user involvement in their care and how users assess their involvement in care decisions.^37^ Indeed, we analyzed two cases before and after the introduction of new guidelines, standards, and policies to make user involvement a core dimension of mental healthcare delivery in Canada.

###  Case Selection

 Cases refer to the clinical teams that deliver community care treatments for users with a severe mental illness. The two cases were selected based on the most-similar case selection procedure described by Gerring.^38^ The pair of cases were similar in all aspects except for the variable of interest, which was the integration of an evidence-based model in mental health that emphasized user recovery. The clinical teams had comparable characteristics in that they delivered outpatient care in mental health, followed-up with users with severe and chronic mental illness, and were located in rural or semi-rural HCOs in the province of Quebec (Canada).

###  Data Collection Methods

 This mixed-methods multiple case study was based on both qualitative and quantitative data that had been collected from 2015 to 2017.

 In all, 25 face-to-face interviews with clinical, mid and top-level managers were conducted (14 for case 1 and 11 for case 2) and 4 focus groups were held with the providers (2 for case 1 and 2 for case 2). The interviews and focus groups were carried out during two periods of data collection (2015 and 2017). Each interview lasted between 45 and 60 minutes and the focus-groups were for 90 or 120 minutes. Recruited managers were involved at different levels of governance in quality management or mental health. At the clinical level, all providers were recruited with the support of the clinical manager. Supplementary file 1 provides more details about the participants and the collected data for the two periods. We also continuously collected clinical and management documents related to user involvement in care at each site. All interviews (Supplementary file 2**)** and focus groups (Supplementary file 3) were conducted and transcribed in French and then translated into English. In addition, a patient-reported experience measure was used to evaluate the users’ perceptions of their involvement in care decisions. The survey contained general demographic questions (eg, age, gender, level of education) and eight items from the dyadic OPTION scale that measures the extent to which providers involve users in decision-making from the user’s perspective.^39,40^ A convenience sample approach was used and all users receiving care and services from the two clinical teams were invited to participate in the survey. The users were recruited in March 2017. Eligible users were adults (18 years and older), followed by the team at the time of data collection, and willing to participate. To administrate the survey, three members of the research team went on-site during a day when recovery services were offered to users. Providers were not present at the time users completed the survey, and team members assisted users in filling out the survey as necessary.

###  Data Analysis

 The qualitative data were analyzed using a hybrid deductive and inductive approach.^41^ Successive phases that combined a deductive and inductive analysis were followed^41^ using QDA Miner Lite software: (1) codification and categorization of the data based on a priori template of codes^42^; (2) identification of new codes and categories following a data-driven analysis^43^; and (3) formulation and verification of the findings with key participants. The first three interviews were coded independently by two reviewers (NC, MPP). Divergent codifications were discussed until both reviewers reached a consensus leading to the final categories and codes.Supplementary file 4 presents the initial categories that were based on our conceptual framework and the new categories and codes that were derived from the inductive analysis of the data.

 The quantitative data were analyzed using SPSS software. Any returned surveys that were missing one or more responses were excluded from the analysis. For each sample (cases 1 and 2), we performed descriptive statistics, calculating mean scores, standard deviations, the percentage of highly involved users for each item, and the overall score of the 8-item scale. Scores were compared between the two samples (cases 1 and 2), and t-tests for independent samples were computed to analyze the differences in scores across the samples.

## Results

 Our findings are presented in three different sections. The first section provides a brief description of the two cases (Table 1). The second section answers questions 1 and 2 and presents the analysis of the key practices and factors that contribute to the enhancement of users’ involvement in care. Finally, the third section answers question 3 and describes the users’ assessment of their involvement in decision-making.

**Table 1:** Summary Profile of the Cases

**Characteristics of the Cases**	**Case 1**	**Case 2**
Type of HCO	HCO 1 Integrated university health and social services center	HCO 2 Integrated health and social services center
Location	Rural setting	Semi-rural setting
Evidence-based model in mental health	No	Yes Assertive treatment model
Clinical setting	Outpatient, community care	Outpatient, community care
Clinical team	Acute day hospital clinic	Assertive community treatment
Composition of the clinical team	Program manager and eight providers including psychiatrists, psychologists, psychoeducation workers, occupational therapist, and nurses	Program manager and nine providers including psychologists, social workers, psychoeducators, and nurses

Abbreviation: HCO, healthcare organization.

###  1. Presentation of the Cases

####  Case 1. Acute Day Hospital Clinic

 The acute day hospital team is an interdisciplinary team offering outpatient care for users with acute mental health issues, such as psychotic, emotional, or personality disorders. Services delivered include assessment, psychiatric services, follow-up, and rehabilitation activities. The team is composed of providers from different training such as psychiatry, psychology, psychoeducation, nursing, and occupational therapy. The users’ follow-up consists of two months, with the intention to avoid hospitalization or shorten the stay in a hospital psychiatric care unit by supporting users when facing a psychotic episode. A program manager is in charge of ensuring the adoption of best clinical practices by the team.

####  Case 2. Assertive Community Treatment

 The assertive community treatment team is an interdisciplinary team that provides intensive, comprehensive, community-based treatment, rehabilitation, and support to users with severe and chronic mental health issues such as schizophrenia, psychotic disorders, schizoaffective disorders, and bipolar disorders. Since 2015, the team has introduced an evidence-based model of care in mental health: the assertive community treatment.^44^ To obtain and maintain the accreditation as an assertive community treatment, the team has to comply with several criteria, including in terms of user recovery and shared decision-making. The team is composed of providers whose training includes social work, rehabilitation, psychoeducation, and nursing. Although the team does not have dedicated psychiatrists, the offered services include initial and continuous assessments, case management, employment and housing assistance, family support and education, substance abuse services, and other rehabilitation services for promoting user recovery and empowerment. The follow-up of users is generally between four and six months, and each user has a key-pivot provider who is responsible for the follow-up, and for communicating and coordinating with other team providers. A program manager ensures the adoption of assertive community treatment best practices by the team.

###  2. Proximity governance: Towards Collaborative Practices With Mental Health Users 

 In the two cases, we identified that proximity governance corresponds to both managerial practices and clinical practices that enhance mental health user involvement in care decisions. Following the data-driven analysis, several key managerial and clinical practices for user involvement were identified as well as challenges encountered by providers. Those key practices derive from the initial category “audit” that refers to the methods and tools that ensure the adoption of best practices or standards related to user involvement (see Conceptual Framework). A synthesis of the main practices and challenges related to mental health user involvement is presented in Table 2.

**Table 2:** Synthesis of the Managerial, Clinical Practices and Challenges Associated With Mental Health User Involvement in Care

**Managerial Practices**	**Provider Practices and Challenges Encountered**
Revising of care protocols Strengthening providers’ knowledge of best practices Facilitating coordination of care around family and user’s needs Integrating PSWs in clinical teams	Involving users in the care planning process Inviting users to share their passions, strengths and life goals Supporting users in defining their recovery-oriented objectives (personal, occupational and relational) Challenges encountered Involving new users and users facing acute crises Defining recovery-based objectives with users Absence of psychiatrists to ensure user involvement in treatment decisions

Abbreviation: PSWs, peer-support workers.

####  Managerial Practices for User Involvement

 In both cases, the role of the program managers was central in encouraging and ensuring the comprehensive adoption of best clinical practices related to user involvement by the teams, as required by Accreditation Canada and the MSSS.

####  Revising care Protocols

 In both teams, the managers established an action plan to ensure that care protocols are revised with an integrated recovery-oriented care approach and users’ involvement in care plans with the providers’ practices.

*“It’s part of my job to show the team what to do and how to update their practices. So I made an action plan, we are updating the protocols, the ways we are practicing with users because the recovery is clearly positioned within our organization as a priority”* (Program manager, case 1).

####  Strengthening Providers’ Knowledge of Best Practices

 In case 1, the manager encouraged providers to be part of a new, continuous improvement committee on accreditation standards. The committee brings together mental health program managers and providers to receive updates on mental health standards and it aims to develop strategies to meet specific standards related to user involvement in care.

####  Facilitating Coordination of Care Around Family and User’s Needs

 In case 2, the manager tried to improve the coordination of care around family and users’ needs using a centralized tool for team coordination. This tool contains important weekly monitored information on users, including the care plan, the recovery-based objectives, and the follow-up contacts with users. Based on the recovery-approach, the program manager has worked to improve the involvement of the users’ families and close relatives. If the user agrees, providers establish regular contact with close relatives, at least monthly. Both the manager and providers have reported that the involvement of relatives is crucial to engage users in ongoing treatment or to establish a link in situations of isolation or withdrawal.

####  Integrating Peer-Support Workers in Clinical Teams

 While the MSSS recommends peer support to enhance users’ involvement and to promote recovery, the two clinical teams have not yet integrated a peer-support worker (PSW). In both cases, the program managers believe that PSWs could play a complementary role to the providers, by offering ongoing support to the user regarding his/her recovery objectives, encourage users to be more compliant with their medications, and help de-stigmatize mental illness among providers. However, in case 1, the integration of PSWs raises an issue about a clear definition of their role and responsibilities in the clinical team. The program manager has reported that some professional unions and providers can be reluctant to work with PSWs.

*“We know that we have a lot to do with professional unions to hire a PSW because they are afraid that he’ll take the place of a provider. We have to demystify the role of the PSW and demonstrate that he can play an important role in the team. The most difficult people to convince are health professionals”* (Program manager, case 1).

 In case 2, the manager has started the process of recruiting and integrating a PSW in the team. To that end, she has demonstrated the role and responsibilities of PSWs and their added value from collaborating with them from a recovery-based perspective. The program manager has also described her experience with another team she manages when she introduced a PSW. The PSWs help to support users in their recovery process and objectives, for example, by working in recovery groups and helping the team to manage difficult situations when a user might refuse treatment.

*“The priority project for me is to integrate a PSW who will be helping the team to work towards recovery and help us to approach and speak to users when they refuse their treatments. It’s part of the National Center of Excellence in Mental Health recommendations, but I really believe in the role of PSWs since I introduced them before in another team, it was two years ago. It has been a very helpful support for the team and the users as well. It was necessary to prepare the group before, to make them understand the role of PSWs” *(Program manager, case 2).

####  Clinical Practices for User Involvement

 In both cases, the role of providers was essential to ensure that users are involved in the care planning process. Providers also reported three main challenges associated with user involvement in care and treatment decisions.

####  Involving Users in the Care Planning Process

 Following the MSSS and accreditation requirements in 2015, the two clinical teams oriented their practice toward recovery and a collaborative care approach based on user autonomy and empowerment. Providers of both teams encourage and support the users to be engaged in developing their care plan. Users are first invited to identify their passions, strengths and life goals, then supported in defining three types of recovery-based objectives: personal, occupational, and relational. In both cases, the involvement of the user in his/her care plan always takes place during the first weekly consultations/visits with the pivot provider (psychiatrist or psychologist) in charge of the user’s follow-up. The care plan is then presented to the interdisciplinary team and in case 2, to the psychiatrist outside the team who ensures the user’s follow-up. However, in both teams, the user is also not involved during case discussions during interdisciplinary meetings. In case 1, the program manager and providers have reported the time constraints for integrating users into interdisciplinary meetings.

####  The Challenge of Involving New Users and Users Facing Acute Crises

 While providers have adopted a collaborative approach with users, they have also reported issues concerning user involvement. In case 1, the manager has raised the difficulty in involving users facing an acute crisis who have to be hospitalized. For psychiatrists in the acute day hospital, it can be difficult to engage users in their care plan in this kind of situation. During an acute crisis, they always inform users about medical decisions.

*“There is still resistance from providers, especially when the user is in an acute phase; there is still resistance to involve the user in decisions at this moment. Sitting with the user to build his plan while he’s in crisis is problematic, but the user is always aware of what the psychiatrists decided for him. It’s a compromise that doctors offer during acute crises. We do not sit at the table with someone who is extremely confused, and in crisis, it’s not helpful”* (Program manager, case 1).

 Similarly, in case 2, the providers reported that in about 10%-15% of cases, users are opposed to collaboration. The most refractory users are new users who do not see any benefit from assertive community treatment care.

*“Now care plans are systematically done; users before it depended on providers, now it’s a requirement. About 85%-90% are done with users, and about 10% to 15% of users refuse to participate in their plan, resistant users who are often new users. In the beginning, it’s not necessarily obvious for them to participate because they do not know what can be done to help them. We always tell them that they can refuse to participate, but the more they know us, the more they want to get involved” *(Psychoeducator, case 2).

####  The Challenge of Defining Recovery-Based Objectives With Users

 The other challenge reported in both cases is collaborating with users when defining recovery-based objectives. Providers usually want to encourage users to have ambitious goals that may not necessarily meet their expectations and needs. One of the strategies is to try to reach a compromise between what the providers think of the users’ capacities to foster recovery and what users want to achieve in their life.

*“I think one of the risks is the imposition of goals that do not correspond to the needs of the user according to the situation in which they find themselves; for example, if the user is using drugs” *(psychologist, case 1).

####  Absence of Psychiatrists in Clinical Teams to Ensure User Involvement in Treatment Decisions

 Finally, case 2 faced a specific issue related to the absence of dedicated psychiatrists on the team, which hinders the users’ involvement in decisions related to their medication. The presence of psychiatrists is one of the standards of practice for assertive community treatment teams, and their integration into the team is a priority for the program manager. The close collaboration between psychiatrists and other providers could facilitate individualized follow-up based on the users’ needs, especially with regards to adjustments to medication, management of side-effects, and improved access to consultations with psychiatrists.

###  3. System Governance: Organizational and Contextual Factors That Facilitate User Involvement In Mental Health

 In both cases, several organizational and contextual factors have facilitated the involvement of mental health users in their care. Those facilitating factors refer to what is called system governance in our initial conceptual framework (see Conceptual Framework). The data-driven analysis allowed us to identify four specific categories: compliance with an organizational commitment to user involvement, conformity to external requirements for user involvement in mental health, access to specific training on evidence-based practices in mental health and the institutionalization of a culture promoting user involvement.Those new categories derive from the three initial categories presented in our framework: internal and external accountability, training and culture.

####  Compliance With an Organizational Commitment to User Involvement

 For both cases, complying with the HCOs commitment to user involvement helped to introduce and implement a collaborative approach with users. Before the introduction of the MSSS and accreditation requirements for user involvement, the HCOs had implemented a collaborative initiative with users as part of their strategic goals, and the quality departments were in charge of deploying it in both HCOs. In case 1, a patient partnership model, consisting of engaging user advisors in quality improvement activities had been initiated and implemented in 2013. In case 2, the chief executive officer (CEO) decided to implement a user experience model in 2011; this model was based on weekly bedside visits to foster user involvement in quality improvement.

*“We do five bedside visits a week in different sectors and clinical units. Visits are always done by the CEO, myself and the program manager. We ask users to identify strengths but also improvements related to their care and the delivery of services” *(Quality Manager, case 2).

 To comply with the HCO’s strategic goals, several clinical teams in case 1, including teams in mental health, started recruiting and integrating users as part of their quality improvement committees and activities. After the introduction of the MSSS and the accreditation requirements for user involvement in care, the participation of user advisors became widespread in most clinical departments and teams. In case 2, all users, including those receiving care in mental health, are asked about their care experience with providers on a weekly basis. The bedside visits are part of the quality department’s collaboration with the manager in charge of the corresponding visited unit. The program manager provides feedback to the team in terms of good or bad practices that have been identified from the perspective of the users. Bedside visits are used to ensure quality improvement in conjunction with patient-reported experience measures like patient satisfaction and experience surveys.

####  Conformity to External Requirements for User Involvement in Mental Health

 To comply with the MSSS action plan for mental health, 2015-2010, the recovery-oriented approach of care was identified, in both HCOs, as a priority for clinical care by the departments of mental health. Furthermore, case 2 had to conform with the directive of the Mental Health Department of the MSSS, as the team must ensure that care delivery meets the standards of practice recognized by the assertive community treatment model of care. One of the standards is specific to user involvement, as in using a recovery-centred approach in treatment, involving the user in elaborating the care plan, and integrating PSWs in the team.

*“We must have a care plan made in interdisciplinarity, which includes the user. When we look at the accreditation visit that is coming, the National Center of Excellence in Mental Health provides 46 criteria; it gives an idea of what is valued by the ministry. One of the aspects is the care plan: are they up to date? Are they made with the user? Having a PSW is also one of the criteria. That’s why I put it in my action plan”* (Program manager, case 2).

####  Access to Specific Training on Evidence-Based Practices in Mental Health

 For several years, both teams benefit from training by the National Center of Excellence in Mental Health, which is part of the mental health department of the MSSS. Its mandate is to help teams develop clinical best practices. Since the adoption of the action plan in mental health, the teams have received training for the recovery-oriented care approach, which emphasizes user involvement in his/her care plan.

*“We have extensive training provided by the National Center of Excellence in Mental Health, yes. All team providers have been trained by the center”* (Program manager, case 1).

 For case 2, providers receive specific training each year to follow the best standards of practice specific to the assertive community treatment model of care, which covers the recovery-oriented and user-involvement approaches. Furthermore, the program manager organized a one-day training session provided by the Quebec Association for Psychosocial Rehabilitation to prepare the integration of a PSW in the team. The training included a presentation of the philosophy and objectives of peer-support in mental health and the role and responsibilities of PSWs on the team. The program manager reported that the training was a necessary step before integrating a PSW because it helped to break taboos and prejudices around the role of the PSW.

*“It is necessary to prepare the team so that they understand the role of PSWs. We give a mini-training to abolish taboos. This training is provided by the AQRP (Québec Association of Psychosocial Rehabilitation), which accompanies the peer-support movement. I had introduced the PSW project to the team two years ago. The team will be ready soon to integrate a PSW” *(Program manager, case 2).

####  Institutionalization of a Culture Promoting User Involvement

 For several years, both HCOs have benefited from collaborative approaches that were implemented at the organizational level and which are part of the core values of the HCOs. In the HCO 1, the integration of user advisors has not resulted in projects for improving user involvement in direct care since improvement projects have been focused on improving the delivery of services based on user needs. Nevertheless, providers have reported that this initiative has helped to destigmatize users by collaborating with them regularly in quality improvement projects and has helped to develop a culture of partnership with users within the team. The CEO and the quality department ensure the promotion of patient partnership in care and services with program managers and providers. The principles and benefits of such approaches are regularly presented at departmental meetings and to medical committees like the Council of Physicians, Dentists, and Pharmacists.

*“Every two months, I meet with the quality department, the clinical directors who are managers and the medical co-directors who are physicians. I also attend most of the meetings of the CMDP (Council of Physicians, Dentists, and Pharmacists). I have many opportunities to present our approach to partnership with users” *(CEO, case 1).

 In the HCO 2, the user experience approach has been used to develop a culture of user-centred care and services. Following bedside visits, “model employees” who have been recognized by users to have good practices are acknowledged by the quality department. The aim of the recognition is to encourage good practices and collaborative practices with users. In case 2, at the clinical level, the program manager changed the ways of involving users in their recovery plan, specifically for employment. One of the changes was related to the employability of users who are not on the track of recovery. The innovative practice is to support the users to be employed while they are still taking drugs, or when they are not compliant with their medications. Employment becomes a way to promote recovery rather than the outcome of the recovery process. The program manager has organized training for providers who work on employability to support them in this innovative practice.

####  “I have two providers who work on employability and who have been trained on that. This training challenges many practices because usually, we think that a person has to stop consuming drugs or alcohol, to take his/her treatments to get ready to work. But now we want to introduce a new way of thinking employability of users. It’s the opposite way of thinking; the person consumes, does not take his/her medication. We want to challenge the old ways of thinking and practicing in our team” (Program manager, case 2).

###  4. Mental Health Users’ Perceptions of Their Involvement in Care Decisions

 The data-set contained 46 user respondents (22 in case 1 and 24 in case 2). The response rate was 23% for case 1 and 24 % for case 2. In case 1, most users were women (62%), whereas in case 2, most were men (71%). All age groups are represented except for users of 30 to 39 years of age in case 1. In cases 1 and 2, most users were under 50 years of age (64% in case 1; 67% in case 2). In both cases, most users had completed secondary school (57% in case 1; 52% in case 2). The demographic characteristics of the users are summarized in Table 3.

**Table 3:** Demographic Characteristics of the Two Samples (Gender, Age, and Education) Showing the Percentages of Respondents

	**Case 1 (ADH** ^a^ **)**	**Case 2 (ACT** ^b^ **)**
Gender, No. (%)	n = 21	n = 24
Men	8 (38.1)	17 (70.8)
Women	13 (61.9)	7 (29.2)
Age, No. (%)	n = 22	n = 24
20-29	5 (22.7)	3 (12.5)
30-39	0 (0.0)	8 (33.3)
40-49	9 (40.9)	5 (20.8)
50-59	6 (27.3)	3 (12.5)
60 and older	2 (9.1)	5 (20.8)
Education, No. (%)	n = 21	n = 23
Primary	1 (4.8)	8 (34.8)
Secondary	12 (57.1)	12 (52.2)
College	6 (28.6)	2 (8.7)
University	2 (9.5)	1 (4.4)

Abbreviations: ADH, acute day hospital; ACT, assertive community treatment. ^a^Case 1 corresponds to the sample of users receiving care from the ADH team. ^b^Case 2 refers to the sample of users receiving care from the ACT team.

 The mean score for the 8-item scale was 23.6 for case 1 and 25 for case 2 (maximum of 32), indicating a high degree of perceived involvement in decision-making (Table 4).

**Table 4:** Descriptive Statistics of the Overall Score of User Involvement (8-Item Scale) in Decision-Making

	**Case 1 (ADH)** **(n = 22)**	**Case 2 (ACT)** **(n = 23** ^a^ **)**
Mean score	23.6	25.0
Standard deviation	6.3	4.4
Range (min-max)^b^	12-31	17-32

Abbreviations: ADH, acute day hospital; ACT, assertive community treatment. ^a^One observation was excluded from the analysis because of more than one missing value on the scale. ^b^Possible range was 8-32.

 As shown in Table 3, the two samples represented different demographic characteristics. To assess whether or not the characteristics were correlated with the overall score, we computed the Spearman correlation coefficients for the total score with gender, age, and education (Table 5). Weak and non-significant correlations (*P*> .05) were found for age and overall score (Spearman rho = 0.18), gender and total score (rho = 0.18), and education and overall score (rho = -0.05). The Spearman correlation coefficients and the *P* values suggested weak correlations between the demographic characteristics and the overall score so that the observed differences in the scores across the two cases were not driven by the distribution of characteristics.

**Table 5:** Spearman Correlations Coefficients (and *P* Values) for the Overall Scores and Gender, Age, and Education

	**Overall Scores** **User Involvement in Decision-Making**	***P*** ** Values**
Gender	0.18	.23
Age	0.18	.23
Education	-0.05	.73

 Since we included eight items of the 12-item dyadic OPTION scale, we computed the reliability of the 8-item scale. Cronbach α was 0.87, suggesting high reliability of the scale.

Table 6 assesses user involvement in decision-making for the eight items in each sample (cases 1 and 2), according to the mean scores, standard deviations, and percentage of highly involved users. High involvement scores were computed by grouping the two highest answer modalities (3 = often and 4 = always). An additional table (Supplementary file 5) presents the item scores according to the four answer modalities, and it shows the wording of questions used in the scale. In both cases, a high proportion of users reported a high degree of perceived involvement in decisions. They felt that they were carefully listened to by their providers when they spoke of their problems (82% of users in case 1 and 100% of users in case 2), involved in discussions about the advantages and disadvantages of options (74% in case 1 and 77% in case 2) and invited to speak of their ideas or expectations to manage problems (77% in case 1 and 82% in case 2). Only item 3 (different types of information used to present options) had a low degree of perceived involvement among users (55% of users in case 1 and 27% of users in case 2). As shown in Table 6, users in case 2 had the highest degree of perceived involvement for all items except for item 3. We could see even small differences in the degree of perceived involvement across the two cases for items 1, 2, 3, 6, and 7. The largest differences in perceived involvement were seen in item 3 (different types of information used to present options) and item 7 (all information explained to ensure understanding). Interestingly, a high proportion of users in case 2 felt highly informed to facilitate their understanding (87.5% of users) while a low percentage of users reported being informed through different types of information (27% of users).

**Table 6:** User Assessment of Their Involvement in Decision-Making by Items of the Dyadic OPTION Scale (n = 8 Items)

	**Mean Score (SD)**		**Percentage of High Degree of Involvement** ^a^		**N**	
	**Case 1**	**Case 2**	**Case 1**	**Case 2**	**Case 1**	**Case 2**
1. Problem(s) listened carefully	3.45 (0.80)	3.71 (0.46)	81.8	100.0	22	24
2. Several options presented to deal with the problem(s)	2.82 (1.14)	3.22 (0.85)	63.6	82.6	22	23
3. Different types of information used to present options	2.45 (1.10)	2.00 (1.15)	54.6	27.3	22	22
4. Advantages and disadvantages of different options discussed	3.09 (0.97)	3.13 (1.18)	77.3	73.9	22	23
5. Users’ ideas or expectations explored	3.05 (0.84)	3.39 (0.99)	77.3	82.6	22	23
6. Users’ concerns or worries explored	2.95 (0.99)	3.24 (0.94)	68.2	85.7	22	21
7. All information explained to ensure understanding	3.09 (1.06)	3.46 (0.72)	68.2	87.5	22	24
8. Time and opportunities to ask questions	2.73 (1.20)	3.13 (1.03)	59.1	70.8	22	24

Abbreviation: SD, standard deviation. ^a^To compute high involvement scores, the two highest answer modalities (3 = often and 4 = always) were grouped into one variable named “high degree of involvement.”

 To test whether or not the differences in scores observed across cases were statistically significant, we computed independent sample t-tests on SPSS. Non-significant differences were found (*P*> .05). These results can be explained by the low number of observations in each sample (n = 22 in case 1 and n = 24 in case 2).

## Discussion

###  Main Contributions of This Paper

 To the best of our knowledge, this is the first research that applies the concept of clinical governance to understand how managerial and clinical practices, and organizational and contextual factors, can enhance the involvement of mental health users in their care. To date, studies on user involvement in mental health have focused on identifying clinical practices or factors associated with user participation without investigating the interplay between managerial and clinical practices or factors that support user engagement in care. In this study, we also found that mental health users who receive treatment from community care treatment teams have a high degree of perceived involvement in their care decisions. This finding supports the literature on user involvement in mental health, by showing that community mental health teams adopting recovery and collaborative-oriented practices show a high degree of users’ perceived involvement in care decisions.

###  “Proximity Governance”: Providers and Managers’ Practices to Foster Mental Health User Involvement in Care

 From the concept of clinical governance, we can understand the interplay between providers and managers in fostering user involvement in their care. In their everyday work, they develop methods, strategies, and tools to improve user involvement. In both cases, providers used two main methods to improve user involvement in the care planning process. They encouraged users to identify their life goals and strengths and supported users in defining their recovery-oriented objectives. User involvement in decision-making is an essential component of recovery-oriented care in mental health.^24^ User involvement has been conceptualized by different authors^11,12^ along a continuum representing diverse approaches. At one end, users are seen to be passive and uninvolved in their care decisions, whereas, at the opposite end, users fully control their care decisions.^45^ This study has shown that providers invite users to collaborate in their care planning process, but because of the time constraints, users are not integrated with interdisciplinary meetings as full-fledged partners in the team (cases 1 and 2). We also observed that providers engaged users somewhere between the two extremes of involvement in care decisions. Thus, providers seek to encourage users to influence their care decisions and share control in orienting their life project.^45^

 Program managers at the clinical level play a key role in adopting clinical practices related to user involvement. In both cases, clinical managers developed action plans to revise protocols related to user involvement in care plans and facilitate the appropriation of recovery-oriented practices (cases 1 and 2). In case 1, the clinical manager particularly emphasized on strengthening providers’ knowledge of mental health and user involvement best practices. In case 2, the clinical manager developed additional strategies to facilitate better collaboration between users and providers in the care process. They used a centralized tool to coordinate the care around the user’s and the family’s needs, established regular contacts with the user’s family or relatives, and integrated a PSW on the team. Studies have shown that families and relatives living with a person with a mental illness can help providers in various ways, including encouraging and supporting treatments, providing crisis intervention, and providing information about the context of the user’s life.^46^ The introduction of PSWs in health teams is in line with studies concluding that peer-support may enhance the involvement of users in their care and recovery processes.^47^ To deal with some of the issues involved with peer-support, clinical managers need to demystify the roles and responsibilities of PSWs to benefit the providers.^48,49^ Interestingly, in case 2, the program manager considers the introduction of a PSW as a way to enhance the users’ adherence to treatments, which underlies the idea of recovery as symptom relief and management. In the literature, two representations of recovery are commonly found: a way to restore functioning or a process to deepen wellness.^50,51^ The first representation relies on a clinical model of care, whereas the second focuses on a citizenship model that derives from the mental health user movement that claims users have a right to self-determination and inclusion in the community.^51^

 Our study also confirmed that fully and systematically engaged mental health users can be challenging for providers with regard to their care.^6^ Involving users in their care process planning is not always easy, and for example, those with a severe mental illness who are in an acute crisis tend to be more difficult to engage.^6^ Providers are also challenged to compromise between what they think of a user’s capacities for recovery and the user’s expectations and preferences for recovery.

###  “System Governance”: Contextual and Organizational Factors Enhancing User Involvement in Mental Health

 Beyond the roles of providers and clinical managers, our study implicates the organizational and contextual factors that can facilitate user involvement in their care. The factors are related to compliance with organizational and external commitments and requirements for user involvement, access to specific training on evidence-based practices in mental health, and the institutionalization of a culture promoting user involvement. In one of the HCOs particularly, a recovery approach was integrated as part of the guiding principles of the health team since the accreditation requirements encouraged the team to meet specific standards regarding recovery activities (case 2). In case 1, the HCO was engaged in deploying a partnership in the care and services approach, as part of the strategic goals. Studies on clinical governance have emphasized the need for HCOs to develop a comprehensive strategy and accountability mechanisms in keeping with international and national standards and guidelines of practice.^27,28^ Our findings suggest that the introduction of user involvement initiatives in quality improvement helps to develop a culture of user participation among providers and managers, and creates the impetus for change in clinical and managerial practices. The literature on clinical governance and change implementation in healthcare practice indicates that developing a culture of innovation is a key factor for sustaining the changing practices in HCOs and clinical settings.^27,52^ Finally, in both cases, access to specific training on evidence-based practices in mental health is crucial for developing the knowledge and skills of providers with regards to recovery and collaborative-oriented practices.

###  Mental Health Users’ Perception of Their Involvement in Care on Community Health Teams

 This study demonstrates that mental health users treated in community care treatment teams have a high degree of perceived involvement in their care decisions. We found that the assertive community treatment team (case 2), implementing various strategies to foster user involvement, had a higher proportion of users with a high degree of perceived involvement in decision-making. In both cases, users felt they were particularly carefully listened to by providers when they spoke of their problems and were involved in discussions about the advantages and disadvantages of options or when they discussed their ideas and expectations for managing problems. These findings add to the literature on community mental healthcare and user involvement in mental health, by showing that the teams, when adopting recovery and collaborative-oriented practices with users can facilitate a high degree of user-perceived involvement in care decisions. Interestingly, a large majority of users felt highly informed by providers to ensure their understanding of their options, while few reports having been informed through different types of tools such as user decision aids,^53^ decision boxes^54^ or the Option grid.^55^ Thus, verbal information seems to facilitate users’ understanding of their options without the need to use information tools. This also brings some nuances to the literature on shared decision-making, which concludes that the use of concrete information tools is essential for improving the participation of users in their decisions.^24,53^

###  Study Limitations and Future Research

 Our research was conducted at outpatient mental health settings which tend to develop collaborative-oriented practices and might have a high degree of user perception for involvement in care, compared to acute and inpatient mental health teams. Further research would be interesting to conduct to better understand how user involvement is integrated and supported in acute and inpatient mental health settings and how users perceive their involvement in care. In this research, we collected a small amount of quantitative data on user perception of their involvement in care. The low response rates among users can be explained by the difficulty recruiting users with a severe mental illness who were being treated by the clinical teams at the time of data collection. Only a small number of users were present during on-site rehabilitation activities, which provided an opportunity for the research team to recruit the participants. We are aware that the low response rate limits our ability to generalize the results and the users who completed the survey may have had a better experience with involvement in care than the users who did not.

## Conclusion

 Our study showed that mental health users treated in community care treatment teams have a high degree of perceived involvement in their care decisions. Our finding supports the need to introduce user involvement-oriented practices such as involving users in their care plan, adopting a recovery-approach and integrating PSWs in health teams. Those practices are based on community care treatment models that are likely to improve the users’ experience of their involvement in care. These findings are instructive in a context where user involvement has become a clinical standard in mental healthcare, through accreditation standards and policy recommendations. By looking at keys practices and factors that facilitate user involvement in care, this study can guide HCOs managers and providers for implementing strategies to facilitate user involvement in care within mental health settings.

## Acknowledgments

 We thank the Canadian Institutes of Health Research and the *Fonds de Recherche du Québec - Santé* for their financial support, and the HCOs and participants who were involved in this study. The authors also thank Francine Desbiens who participated in the data collection and Glen Wheeler for his contribution to the editing of this manuscript.

## Ethical issues

 This study was approved by the University of Montreal Health Sciences Research Ethics Committee (certificate #14-127-CERES-D). All interviewees and focus group participants received, read, understood, and signed consent forms before participating in the study. To protect the privacy of mental health users who participated in the study, healthcare providers were not present when users completed the survey nor aware of which users had agreed to take part in the study.

## Competing interests

 Authors declare that they have no competing interests.

## Authors’ contributions

 NC is the main author of the manuscript. Both authors contributed to at least some components of the study and manuscript. NC shaped all aspects of the study design, with feedback from MPP. NC and MPP contributed to the collection of data. NC analyzed all the material with support and feedback from MPP. NC wrote the first draft and MPP gave substantial feedback. Both authors have read and approved the final manuscript.

## Authors’ affiliations

^1^Ingram School of Nursing, McGill University, Montreal, QC, Canada. ^2^McGill University Health Centre, Montreal, QC, Canada. ^3^Department of Health Policy, Management and Evaluation, School of Public Health, University of Montreal, Montreal, QC, Canada.

## Supplementary files


Supplementary file 1. Data collection.

Supplementary file 2. A Selection of Interview Questions With Program/Clinical Managers.

Supplementary file 3. A Selection of Interview Questions With Providers (Focus-Groups).

Supplementary file 4. Initial Categories, Emerging Categories and Codes Deriving From Data Analysis.

Supplementary file 5. Assessment of User Involvement in Decision-Making for the 8-Item Scale According to the Four Answer Modalities.

## References

[1] Crawford MJ, Rutter D, Manley C (2002). Systematic review of involving patients in the planning and development of health care. BMJ.

[2] Trotti A, Colevas AD, Setser A, Basch E (2007). Patient-reported outcomes and the evolution of adverse event reporting in oncology. J Clin Oncol.

[3] Hibbard JH, Greene J, Tusler M (2009). Improving the outcomes of disease management by tailoring care to the patient’s level of activation. Am J Manag Care.

[4] Hibbard JH, Greene J (2013). What the evidence shows about patient activation: better health outcomes and care experiences; fewer data on costs. Health Aff (Millwood).

[5] Bodenheimer T, Lorig K, Holman H, Grumbach K (2002). Patient self-management of chronic disease in primary care. JAMA.

[6] Dixon LB, Holoshitz Y, Nossel I (2016). Treatment engagement of individuals experiencing mental illness: review and update. World Psychiatry.

[7] World Health Organization (WHO). The World Health Report 2001: Mental Health: New Understanding, New Hope. Geneva: WHO; 2001.

[8] Chisholm D, Flisher AJ, Lund C (2007). Scale up services for mental disorders: a call for action. Lancet.

[9] United Nations. Convention on the Rights of Persons with Disabilities. https://www.un.org/development/desa/disabilities/convention-on-the-rights-of-persons-with-disabilities/convention-on-the-rights-of-persons-with-disabilities-2.html. Accessed October 3, 2020. Published 2020.

[10] Grande SW, Faber MJ, Durand MA, Thompson R, Elwyn G (2014). A classification model of patient engagement methods and assessment of their feasibility in real-world settings. Patient Educ Couns.

[11] Carman KL, Dardess P, Maurer M (2013). Patient and family engagement: a framework for understanding the elements and developing interventions and policies. Health Aff (Millwood).

[12] Pomey MP, Flora L, Karazivan P (2015). Le “Montreal model”: enjeux du partenariat relationnel entre patients et professionnels de la santé. Sante Publique.

[13] Charles C, Gafni A, Whelan T (1999). Decision-making in the physician-patient encounter: revisiting the shared treatment decision-making model. Soc Sci Med.

[14] Barry MJ, Edgman-Levitan S (2012). Shared decision making--pinnacle of patient-centered care. N Engl J Med.

[15] Elwyn G, Frosch D, Thomson R (2012). Shared decision making: a model for clinical practice. J Gen Intern Med.

[16] Flynn D, Knoedler MA, Hess EP (2012). Engaging patients in health care decisions in the emergency department through shared decision-making: a systematic review. Acad Emerg Med.

[17] Légaré F, Witteman HO (2013). Shared decision making: examining key elements and barriers to adoption into routine clinical practice. Health Aff (Millwood).

[18] Gravel K, Légaré F, Graham ID (2006). Barriers and facilitators to implementing shared decision-making in clinical practice: a systematic review of health professionals’ perceptions. Implement Sci.

[19] Pomey MP, Ghadiri DP, Karazivan P, Fernandez N, Clavel N (2015). Patients as partners: a qualitative study of patients’ engagement in their health care. PLoS One.

[20] Karazivan P, Dumez V, Flora L (2015). The patient-as-partner approach in health care: a conceptual framework for a necessary transition. Acad Med.

[21] Mental Health Commission of Canada (2015). Guidelines for Recovery-Oriented Practice.

[22] Jacob KS (2015). Recovery model of mental illness: a complementary approach to psychiatric care. Indian J Psychol Med.

[23] Davidson L, Tondora J, Pavlo AJ, Stanhope V (2017). Shared decision making within the context of recovery-oriented care. Ment Health Rev (Brighton).

[24] Dahlqvist Jönsson P, Schön UK, Rosenberg D, Sandlund M, Svedberg P (2015). Service users’ experiences of participation in decision making in mental health services. J Psychiatr Ment Health Nurs.

[25] Accreditation Canada. Client- And Family-Centered Care in the Qmentum Program. Ottawa: Accreditation Canada; 2015.

[26] Ministère de la Santé et des Services sociaux. Plan d’action en santé mentale 2015-2020. Québec: Gouvernement du Québec; 2015.

[27] Buetow SA, Roland M (1999). Clinical governance: bridging the gap between managerial and clinical approaches to quality of care. Qual Health Care.

[28] Brault I, Roy DA, Denis JL (2008). Introduction à la gouvernance clinique: historique, composantes et conceptualisation renouvelée pour l’amélioration de la qualité et de la performance des organisations de santé. Prat Organ Soins.

[29] Gray C (2005). What is clinical governance?. BMJ.

[30] O’Connor N, Paton M (2008). ‘Governance of’ and ‘Governance by’: implementing a clinical governance framework in an area mental health service. Australas Psychiatry.

[31] Halligan A, Donaldson L (2001). Implementing clinical governance: turning vision into reality. BMJ.

[32] Pomey MP, Denis JL, Contandriopoulos AP (2008). A conceptual framework for analysing clinical governance in healthcare establishments. Prat Organ Soins.

[33] Som CV (2004). Clinical governance: a fresh look at its definition. Clinical Governance: An International Journal.

[34] Scally G, Donaldson LJ (1998). The NHS’s 50 anniversary Clinical governance and the drive for quality improvement in the new NHS in England. BMJ.

[35] Yin RK. Case Study Research: Design and Methods. Thousand Oaks, CA: SAGE Publications; 2009.

[36] Mills A, Durepos G, Wiebe E. Encyclopedia of Case Study Research. Thousand Oaks, CA: SAGE Publications; 2010. 10.4135/9781412957397

[37] Calman L, Brunton L, Molassiotis A (2013). Developing longitudinal qualitative designs: lessons learned and recommendations for health services research. BMC Med Res Methodol.

[38] Gerring J. Case Selection for Case-Study Analysis: Qualitative and Quantitative Techniques. In: Box-Steffensmeier JM, Brady HE, Collier D, eds. The Oxford Handbook of Political Methodology. Oxford: Oxford University Press; 2008.

[39] Elwyn G, Edwards A, Wensing M, Hood K, Atwell C, Grol R (2003). Shared decision making: developing the OPTION scale for measuring patient involvement. Qual Saf Health Care.

[40] Elwyn G, Hutchings H, Edwards A (2005). The OPTION scale: measuring the extent that clinicians involve patients in decision-making tasks. Health Expect.

[41] Fereday J, Muir-Cochrane E (2006). Demonstrating rigor using thematic analysis: a hybrid approach of inductive and deductive coding and theme development. Int J Qual Methods.

[42] Crabtree B, Miller W. Doing Qualitative Research. 2nd ed. SAGE Publications; 2002.

[43] Boyatzis RE. Transforming Qualitative Information: Thematic Analysis and Code Development. Thousand Oaks, CA: SAGE Publications; 1998.

[44] Stein LI, Santos AB. Assertive Community Treatment of Persons with Severe Mental Illness. New York, NY: W. W. Norton & Company; 1998.

[45] Livingston JD, Nijdam-Jones A, Lapsley S, Calderwood C, Brink J (2013). Supporting recovery by improving patient engagement in a forensic mental health hospital: results from a demonstration project. J Am Psychiatr Nurses Assoc.

[46] Canadian Mental Health Association. Family mental health alliance. Caring Together: Families as Partners in the Mental Health and Addiction System. Toronto: Canadian Mental Health Association; 2006.

[47] Repper J, Carter T (2011). A review of the literature on peer support in mental health services. J Ment Health.

[48] Pelletier JF, Tourette-Turgis C (2016). Recovery-oriented medical training: a narrative literature review for the university of recoveryas a new concept of co-learning between patients and (future) healthcare providers. J Community Med Health Educ.

[49] Pelletier JF (2017). Civic recovery mentorship: an online undergraduate medical training program to transform experience into expertise, and attitudes into competencies. Ann Med Health Sci Res.

[50] Hess JZ, Lacasse JR, Harmon J, Williams D, Vierling-Claassen N (2014). “Is there a getting better from this, or not?” examining the meaning and possibility of recovery from mental disorder. Child Youth Serv.

[51] Pelletier JF, Corbière M, Lecomte T (2015). Citizenship and recovery: two intertwined concepts for civic-recovery. BMC Psychiatry.

[52] Scott T, Mannion R, Davies HT, Marshall MN (2003). Implementing culture change in health care: theory and practice. Int J Qual Health Care.

[53] Stacey D, Légaré F, Col NF (2014). Decision aids for people facing health treatment or screening decisions. Cochrane Database Syst Rev.

[54] Giguere A, Légaré F, Grad R (2012). Decision boxes for clinicians to support evidence-based practice and shared decision making: the user experience. Implement Sci.

[55] Elwyn G, Lloyd A, Joseph-Williams N (2013). Option Grids: shared decision making made easier. Patient Educ Couns.

