# Supplementary material for: Clinical Governance to Enhance User Involvement in Care: A Canadian Multiple Case Study in Mental Health
Source: Int J Health Policy Manag. 2020 Nov 7;11(5):658–69. doi: 10.34172/ijhpm.2020.208 (PMC9309928; doi:10.34172/ijhpm.2020.208)
Supplement: Supplementary file 1 — Data collection. [file ijhpm-11-658-s001.pdf]

Supplementary file 1. Data collection

	**Case 1**	**Case 2**
**Interviews**	N=14<br><br>Clinical managers (program managers)<br><br>Mid-level managers<br>(quality department, mental health department)<br><br>Top-level managers (chief executive officers)	N=11<br><br>Clinical manager (program managers)<br><br>Mid-level managers<br>(quality department, mental health department)<br><br>Top-level managers (chief executive officers)
**Focus-groups with providers**	N=2<br>With 8 providers	N=2<br>With 9 providers
**Surveys with users**	N=22	N=24
