# Supplementary material for: Clinical Governance to Enhance User Involvement in Care: A Canadian Multiple Case Study in Mental Health
Source: Int J Health Policy Manag. 2020 Nov 7;11(5):658–69. doi: 10.34172/ijhpm.2020.208 (PMC9309928; doi:10.34172/ijhpm.2020.208)
Supplement: Supplementary file 2 — A Selection of Interview Questions With Program/Clinical Managers. [file ijhpm-11-658-s002.pdf]

Supplementary file 2. A Selection of Interview Questions With Program/Clinical Managers

1. What is your function in the organization?

2. Can you describe your daily role in improving clinical practices within your team?

3. How are users involved in their care? In what way(s) do health providers involve them?

4. In what way(s) do you ensure that users are involved in their care? What are your roles and practices with your team to promote collaborative practices with users?

5. With whom (clinical or administrative department program of care, type of managers, health providers) do you collaborate to strengthen these collaborative practices in care?

6. What factors facilitate user involvement in their care?

7. What are the challenges faced by your team in involving users in their care? How do you, as a manager, respond to these challenges?
