# Supplementary material for: Clinical Governance to Enhance User Involvement in Care: A Canadian Multiple Case Study in Mental Health
Source: Int J Health Policy Manag. 2020 Nov 7;11(5):658–69. doi: 10.34172/ijhpm.2020.208 (PMC9309928; doi:10.34172/ijhpm.2020.208)
Supplement: Supplementary file 3 — A Selection of Interview Questions With Providers (Focus-Groups). [file ijhpm-11-658-s003.pdf]

Supplementary file 3. A Selection of Interview Questions With Providers (Focus-Groups)

1. What is your profession and role within the clinical team?

2. Can you describe your daily role in providing care to the users?

3. How are users involved in their care? In what way(s) do you involve them in the care planning?

4. In what way(s) do you ensure that users are involved in their care? What are your roles and practices regarding user involvement?

5. How do you collaborate with your team (other providers, clinical manager) to strengthen the collaborative practices with users? Are you involved in specific activities or training?

6. What factors facilitate user involvement in their care?

7. What challenges do you face in involving users in their care?
