# Supplementary material for: Clinical Governance to Enhance User Involvement in Care: A Canadian Multiple Case Study in Mental Health
Source: Int J Health Policy Manag. 2020 Nov 7;11(5):658–69. doi: 10.34172/ijhpm.2020.208 (PMC9309928; doi:10.34172/ijhpm.2020.208)
Supplement: Supplementary file 4 — Initial Categories, Emerging Categories and Codes Deriving From Data Analysis. [file ijhpm-11-658-s004.pdf]

Supplementary file 4.

<table>
<tr><th>Initial categories</th><th>Proximity governance</th><th>Audit/quality improvement</th><th>System governance</th><th>Accountability</th><th>Training</th><th>Culture</th></tr>
<tr><td>New categories and sub-categories</td><td colspan="2">Proximity governance
o Managerial practices for user involvement
o Clinical practices for user involvement
o Challenges encountered to involve users</td><td colspan="4">System governance
o Organizational factors facilitating user involvement
o Contextual factors facilitating user involvement</td></tr>
<tr><td>Emerging codes</td><td colspan="2">Managerial practices
o Revision of care protocols
o Strengthening providers’ knowledge of best practices
o Coordination of care around family and user’s needs
o Integration of peer-support workers

Clinical practices
o User involvement in the care planning process
o Challenges encountered by providers</td><td colspan="4">Organizational factors
o Organizational commitment to user involvement
o Institutionalization of a culture promoting user involvement

Contextual factors
o External requirements for user involvement
o Access to training on evidence-based practices in mental health</td></tr>
</table>
