# Supplementary material for: Clinical Governance to Enhance User Involvement in Care: A Canadian Multiple Case Study in Mental Health
Source: Int J Health Policy Manag. 2020 Nov 7;11(5):658–69. doi: 10.34172/ijhpm.2020.208 (PMC9309928; doi:10.34172/ijhpm.2020.208)
Supplement: Supplementary file 5 — Assessment of User Involvement in Decision-Making for the 8-Item Scale According to the Four Answer Modalities. [file ijhpm-11-658-s005.pdf]

**Supplementary file 5.** Assessment of User Involvement in Decision-Making for the 8-Item Scale According to the Four Answer Modalities

| | | **Frequency (%)** | |
|---|---|---|---|
| | | **Case 1** | **Case 2** |
| 1. Problem(s) listened carefully | *Never* | 0 | 0 |
| | *Sometimes* | 4 (18.2) | 0 |
| | *Often* | 4 (18.2) | 7 (29.2) |
| | *Always* | 14 (63.6) | 12 (70.8) |
| | | *n=22* | *n=24* |
| 2. Several options presented to deal with problem(s) | *Never* | 4 (18.2) | 1 (4.3) |
| | *Sometimes* | 4 (18.2) | 3 (13.0) |
| | *Often* | 6 (27.3) | 9 (39.1) |
| | *Always* | 8 (36.3) | 10 (43.5) |
| | | *n=22* | *n=23* |
| 3. Different types of information used to present options | *Never* | 6 (27.3) | 10 (45.4) |
| | *Sometimes* | 4 (18.2) | 6 (27.3) |
| | *Often* | 8 (36.4) | 2 (9.1) |
| | *Always* | 4 (18.2) | 4 (18.2) |
| | | *n=22* | *n=22* |
| 4. Advantages and disadvantages of different options discussed | *Never* | 2 (9.1) | 4 (17.4) |
| | *Sometimes* | 3 (13.6) | 2 (8.7) |
| | *Often* | 8 (36.4) | 4 (17.4) |
| | *Always* | 9 (40.9) | 13 (56.5) |
| | | *n=22* | *n=23* |
| 5. Users' ideas or expectations explored | *Never* | 1 (4.5) | 2 (8.7) |
| | *Sometimes* | 4 (18.2) | 2 (8.7) |
| | *Often* | 10 (45.5) | 4 (17.4) |
| | *Always* | 7 (31.8) | 15 (65.2) |
| | | *n=22* | *n=23* |
| 6. Users' concerns or worries explored | *Never* | 2 (9.1) | 2 (9.5) |
| | *Sometimes* | 5 (22.7) | 1 (4.8) |
| | *Often* | 7 (31.8) | 8 (38.1) |
| | *Always* | 8 (36.4) | 10 (47.6) |
| | | *n=22* | *n=21* |

| | | | |
|---|---|---|---|
| | | | |
| 7. All information explained to ensure understanding | *Never* | 2 (9.1) | 0 |
| | *Sometimes* | 5 (22.7) | 3 (12.5) |
| | *Often* | 4 (18.2) | 7 (29.2) |
| | *Always* | 11 (50.0) | 14 (58.3) |
| | | *n=22* | *n=24* |
| 8. Time and opportunities given to ask questions | *Never* | 5 (22.7) | 2 (8.3) |
| | *Sometimes* | 4 (18.2) | 5 (20.8) |
| | *Often* | 5 (22.7) | 5 (20.8) |
| | *Always* | 8 (36.4) | 12 (50.0) |
| | | *n=22* | *n=24* |

Assessment of user involvement in decision-making for the 8 items of the dyadic OPTION scale, measured on a 4-point Likert scale where 1=never, 2=sometimes, 3=often, 4=always
